# Proteinuria reduction after treatment with miltefosine and allopurinol in dogs naturally infected with leishmaniasis

**DOI:** 10.14202/vetworld.2016.904-908

**Published:** 2016-08-26

**Authors:** Daniela Proverbio, Eva Spada, Giada Bagnagatti de Giorgi, and Roberta Perego

**Affiliations:** Department of Veterinary Medicine, University of Milan, Via Celoria 10, 20133 Milan, Italy

**Keywords:** canine leishmaniasis therapy, proteinuric kidney disease, renal function, urine protein creatinine

## Abstract

**Aim:**

The aim of this study was to evaluate changes in proteinuria in dogs naturally infected with visceral leishmaniasis, following treatment with miltefosine (MLF) and allopurinol.

**Materials and Methods:**

Medical records of 40 dogs with leishmaniasis, treated with 2 mg/kg MLF every 24 h PO and 10 mg/kg allopurinol every 12 h for 28 days were reviewed. 20 dogs were included in the study, and clinical staging was performed following guidelines of the Canine leishmaniasis (CanL) Working Group, and dogs were categorized for proteinuria according to the International Renal Interest Society (IRIS) staging system. Clinical score, indirect fluorescent antibody test titer, serum total protein, gamma globulin (IgG), serum creatinine and urea concentration, and urine protein creatinine ratio (UP/C) were recorded at the time of diagnosis before the start of therapy (D0) and at the end of 28 days of therapy (D28).

**Results:**

Following the CanL Working Group staging, all 20 dogs were classified as the clinical Stage C (Clinical disease) before and after the cycle of treatment. Before the cycle of therapy, dogs were categorized according to the IRIS staging system, as: 9/20 non-proteinuric (NP), 7/20 borderline proteinuric (BP), and 4/20 proteinuric (P). After treatment, 12/20 dogs were NP, 7/20 were BP, and 1/20 was P. There was a significant change in UP/C values before and after one cycle of treatment with MLF. In detail, after 28 days of therapy, 2 of 9 NP dogs became BP, 3 of the 7 BP dogs became NP, and 2 of the 4 P dogs became NP.

**Conclusion:**

This study showed a significant decrease in UP/C values occurred after one cycle of treatment with MLF and allopurinol in dogs naturally affected with CanL. This suggests that MLF does not increase proteinuria, and the use of MLF could be considered for the management of dogs with leishmaniasis, particularly in those with impaired renal function at the time of diagnosis.

## Introduction

Canine leishmaniasis (CanL) is a life-threatening zoonotic disease widely spread throughout the Mediterranean basin and caused by a flagellate protozoan, *Leishmania infantum* [1]. The main clinical findings of CanL are weight loss, muscle atrophy, lymphadenomegaly, dermatological lesions, non-regenerative anemia, serum hyperproteinemia, polyclonal hyperglobulinemia, decreased albumin/globulin ratio, renal azotemia, and persistent renal proteinuria [2]. In dogs with clinical signs of CanL, there is an immune system shift toward a humoral (Th2) response. The activation of B-cells produces high levels of immunoglobulin and production of immune-complexes composed of IgG, IgM, and/or IgA [3,4]. Immune-mediated mechanisms play a pivotal role in the development of renal pathology [5,6].

In dogs with CanL, it is important to monitor proteinuria using the urinary protein creatinine ratio (UP/C), as proteinuria is one of the first signs of renal impairment [2,7]. Moreover, it is essential that drugs used for the treatment of CanL have a low impact on the kidneys, particularly in dogs with impaired renal function at the time of diagnosis [8].

The standard protocol for treatment of CanL has been combination therapy with meglumine antimoniate and allopurinol [9,10]. Miltefosine (MLF) (in combination with allopurinol) is the most commonly used alternative treatment to meglumine antimoniate and the only oral drug for the treatment of CanL [11,12]. Multiple *in vivo* and *in vitro* trials have demonstrated its leishmania killing activity [13], which is effected through disruption of both signaling pathways and cell membrane synthesis, inducing an apoptosis-like cell death. A recent study [8] reported that MLF has limited impact on kidney function in healthy dogs, and no glomerular or tubular lesions were found on kidney biopsies in healthy dogs experimentally treated with MLF. The suggestion is that proteinuria does not increase in dogs treated with MLF. The importance of minimizing renal impairment in dogs with CanL means that the choice of a safe pharmacological treatment is crucial.

The aim of this study was to evaluate changes in proteinuria before and after treatment with MLF and allopurinol in dogs naturally infected with leishmaniasis. In addition, the variations between serum levels of total globulin, total protein (TP), urea, creatinine, and antibodies, before and after treatment, were evaluated.

## Materials and Methods

### Ethical approval

This retrospective study was performed at the Department of Veterinary Sciences for Health, Animal Production and Food Safety (VESPA) of the University of Milan, and it did not include experimental animals, and it was performed on client-owned dogs referred to our institution for therapeutic purpose. All owners gave informed consent for treatments, measurements, and for data recording. The study was carried out in accordance with Italian law (DL 14^th^ March 2014 n.26) and Europe Union legislation covering the use of animal for the scientific purpose (“Animal Scientific Procedures Act” 63/2010/EU) and with the Institutional Ethical Guidelines.

### Inclusion criteria and clinical procedures

A retrospective review was carried out of the medical records of 40 dogs with confirmed leishmaniasis presented at the Internal Medicine Service of the Department of Health, Animal Science and Food Safety, University of Milan, Italy, between 2008 and 2014 that had received a leishmanicidal therapy with 2 mg/kg q 24 h PO of MLF in combination with allopurinol at 10 mg/kg q 12 h for 28 days PO.

### Data collection

Dogs were eligible for inclusion in the study if they fulfilled the following criteria: Physical examination at the first consultation and the end of the cycle of MLF treatment, with severity of signs referable to CanL scored on the basis of clinical score according to the signs reported in a previous study [14]; assigning a value from 0 to 3 to each parameter, depending on the severity of each sign, with a maximum achievable score of 86/86 [15]; a diagnosis of leishmaniasis established by clinicopathologic abnormalities, positive serology for *L. infantum* using immunofluorescence antibody (IFA) test and cytologic identification of leishmania amastigotes, or detection of parasite DNA using polymerase chain reaction in either lymph node or bone marrow aspirates according to diagnostic criteria previously described [2]; hematological and serum biochemical blood examination, IFA test for CanL and complete urinalysis, on samples collected by cystocentesis, including UP/C, at the time of the diagnosis and at the end of the cycle of MLF treatment; residence in a CanL non-endemic area of Northern Italy (to avoid any possible reinfection).

All dogs with other neoplastic, degenerative, inflammatory, endocrine, immunologic, and genetic diseases potentially associated with proteinuria were excluded. Dogs were also excluded if they had concomitant infectious diseases (e.g. babesiosis, ehrlichiosis, and dirofilariasis) diagnosed by parasitological or serological examinations. Any dogs receiving additional medical treatment, including nephrotoxic drugs and those that might change proteinuria, e.g. steroids and angiotensin-converting enzyme inhibitors, were also excluded.

The following information was extracted from the medical records of each dog: Signalment (age, sex, breed, and provenance/travel history), clinical score with clinicopathological signs of CanL, indirect fluorescent antibody test (IFAT) titer, serum TP, gamma globulin (IgG), serum creatinine and urea concentrations, and UP/C. Data were recorded at the time of the diagnosis before the start of therapy (D0) and at the end of the 28 days treatment period (D28).

Dogs were classified on the basis of stage of disease following the clinical staging of the CanL Working Group [16]. Dogs were considered to be Stage A (exposed) when they had no clinical signs, tested negative for parasites, and had a low antibody titer; in Stage B (infected), when they had no clinical signs, positive identification of parasite, and had a low antibody titer; in Stage C (clinical disease), when they had clinical signs and/or clinicopathological abnormalities associated with leishmaniasis, positive identification of parasite and/or antibody titer >4 times the reference value; in Stage D (severe clinical disease), when they had all signs of Stage C and severe concomitant signs such as proteinuria, severe kidney disease or severe ophthalmic or joint disease. Dogs were also classified on the basis of UP/C value according to the International Renal Interest Society (IRIS) guidelines (http://www.iris-kidney.com, 2016-01-20). Dogs were considered: Non-proteinuric (NP) when UP/C was <0.2; borderline proteinuric (BP) when UP/C was 0.2 to 0.5, and proteinuric (P) when UP/C was more than 0.5.

### Statistical analysis

Data statistical analyses were performed using commercial statistical software (MedCalc, v.12.3.0). The distribution of data was assessed using descriptive statistics Kolmogorov–Smirnov test. Parameters before and after therapy were compared using a paired t-test when the data were normally distributed, or the non-parametric Wilcoxon matched paired-test when the data were not normally distributed. The level of significance was set at p<0.05.

## Results

A total of 20/40 dogs met the criteria for inclusion in the study. Ages of these 20 dogs ranged from 1 to 14 years. 14 were intact males and 6 were female (4 neutered), 14 were X-breeds and 6 were purebreds.

Following the staging of CanL as previous described [16], before and after the cycle of treatment, all 20 dogs were classified as clinical Stage C (clinically diseased). Mean, median, and confidence interval 95% and p-value of the clinical score, IFAT titer, TP, gamma globulin, creatinine, urea, UP/C before and after therapy for the 20 dogs treated are reported in Table-1. The value of UP/C in each subject before and after the treatment is reported in Table-2.

**Table-1:** Mean, median, 95% CI, interquartile interval and P value of clinical score, IFAT titer, TP (g/dl), IgG (%), creatinine and urea (mg/dl), and UP/C in 20 dogs before and after 28 days of therapy.

Parameter	D0	D28	p value
Clinical score			
NV: 0 Mean (95% CI)	5.1 (3.1-7.09)	2.6 (1.52-3.77)	p=0.0046
IFAT titer			
NV: Titer<1:80 (minmax)	160-1280 (320-640)	80-1280 (240-640)	p=0.2661
TP			
NV: 68 g/dl Mean (95% CI)	8 (7.55-8.45)	7.6 (7.24-7.96)	p=0.0730
IgG			
NV: 5.39.9% Mean (95% CI)	21.36 (16.89-25.83)	19.06 (14.75-23.36)	p=0.1009
Creatinine			
NV<1.2 mg/dl (Median IIII interquartile interval)	0.91 (0.8-1.05)	0.91 (0.7-1.1)	p=0.8077
Urea			
NV: 1545 mg/dl (Median IIII interquartile interval)	40.75 (29-45.5)	38.55 (29.5-44)	p=0.4171
UP/C			
NP: <0.2 BP: 0.20.5 P: >0.5 (Median I-III interquartile interval)	0.35 (0.1-0.7)	0.26 (0.1-0.4)	p=0.016

NV: Normal value, NP: Non-proteinuric, BP: Borderline proteinuric, P: Proteinuric, CI: Confidence interval 95%, TP: Total protein, IFAT: Indirect fluorescent antibody test, UP/C: Urine protein creatinine, IgG: Gamma globulin

**Table-2:** UP/C value in 20 dogs before and after the treatment.

Dog	D0 UP/C value	D0 UP/C value	Dog	D0 UP/C value	D0 UP/C value
1	0.9 P	0.4 BP	10	0.1 NP	0.1 NP
2	0.3 BP	0.2 BP	11	0.1 NP	0.1 NP
3	0.2 BP	0.1 NP	12	0.9 P	2 P
4	0.1 NP	0.1 NP	13	0.1 NP	0.4 BP
5	0.8 P	0.1 NP	14	0.1 NP	0.1 NP
6	0 NP	0.1 NP	15	0.1 NP	0 NP
7	0.2 BP	0.1 NP	16	0.2 BP	0.3 BP
6	0.5 BP	0.3 BP	17	1 P	0 NP
8	0.1 NP	0.1 NP	18	0.9 P	0.4 BP
9	0.4 BP	0.4 BP	19	0.3 BP	0.2 BP
10	0.1 NP	0.1 NP	20	0.2 BP	0.1 NP

P: Proteinuric, BP: Borderline proteinuric, NP: Nonproteinuric, UP/C: Urine protein creatinine

According to IRIS staging system before the cycle of MLF and allopurinol, 9/20 were NP, 7/20 were BP, and 4/20 were P. After treatment, 12/20 dogs were NP, 7/20 were BP, and 1/20 was P. In particular, 2 of 9 NP dogs became BP, 3 of 7 dogs with BP became NP, and the other 3 remained BP, but P decreased; 2 of 4 P dogs became NP.

Except the value of UP/C, there were no significant differences between any parameters before and after the cycle of therapy with miltefosine and allopurinol. All dogs demonstrated clinical response to therapy, with significant improvement of the clinical score and a reduction of UP/C mean value.

## Discussion

The glomerulonephropathy that characterizes CanL is the result of a glomerular deposition of circulating immune complexes from persistent antigenemia [17]. The consequent activation of the complement system causes injury to the glomerular capillaries and mesangium [17]. Immune complex deposition in glomeruli causes glomerulonephritis and proteinuria [18]. Even though some studies report new markers of renal damage in dogs with CanL, such as ferritin and cystatin C concentrations [19] and urinary gamma-glutamyl transferase value [20], proteinuria is still considered the earliest sign of kidney injury in CanL [2]. There is strong evidence that proteinuria is a risk factor for the development and progression of renal failure which is the most severe complication of CanL and carries a poor prognosis [17,18,21]. Furthermore, a recent study highlights the correlation between elevated blood pressure and increased UP/C in dogs with CanL [22]. So, it is important that any drug used for the treatment of CanL does not alter renal function and does not increase the magnitude of proteinuria. Some studies in dogs have reported the short-term efficacy of MLF therapy in association with allopurinol and reported that this combination is a safe, convenient, and effective alternative treatment option for CanL, producing only mild (and self-limiting) side effects [23-26]. A controlled study [20] has evaluated the effectiveness and safety of MLF and allopurinol therapy in CanL reporting that renal and hepatic parameters were not affected by the therapy.

Bianciardi *et al*. [8] evaluated the pharmacological and toxicological effects of meglumine antimoniate and MLF in healthy dogs experimentally treated and submitted to renal biopsy after 27 days of treatment, showing that in the MLF-treated group there were no ultrastructural lesions, while tubular damage was found in the meglumine antimoniate treated group.

The results of our study are in accordance with the previous report of Bianciardi *et al*. [8] since we found a significant reduction of proteinuria after 28 days of therapy with miltefosine and allopurinol in dogs naturally affected with CanL. In fact, after 28 days of therapy, 2 of 4 P dogs became NP and 3 of 7 BP dogs became NP. All dogs receiving any treatment to reduce proteinuria, other than allopurinol, were excluded from the study to isolate the effects of MLF.

All dogs in this study demonstrated clinical response to therapy, with significant improvement of the clinical score; this is in agreement with results of previous studies [23-26]. There was no statistically significant difference between D0 and D28 for any of the measured parameters except the value of UP/C. Moreover, there was a reduction in the mean percentage of IgG in the mean concentration of TP and the mean value of IFAT titer. Despite the improvement in the average value of these parameters, they remained above normal limits. This finding does not indicate a lack of efficacy of treatment because the therapeutic effect of MLF persists after the 28 days of therapy. This long-lasting activity of MLF may be attributed to its long elimination half-life in canines, leading to a high level of drug accumulation with persistence of therapeutic effects after the treatment period [25]. In addition, CanL affects multiple organs and systems, resulting in polymorphic perturbations in hematologic and biochemical parameters that might require different times to resolve.

We would like to emphasize that recent studies [16,26] report that the effects of meglumine antimoniate in combination with allopurinol are better than MLF with allopurinol for treatment of leishmaniasis in dogs because there was a decrease in the incidence of disease recurrence in dogs treated with meglumine antimoniate plus allopurinol. The clinician must carefully assess the relationship between safety and efficacy when selecting therapy. The results of this study provide a clinically relevant finding that could aid the clinical management of proteinuric dogs with leishmaniasis and may help to establish a treatment regime that reduces the progression of renal disease. Because of the small number of dogs included in this study and the short period of follow–up, results need to be confirmed in a larger study including a larger number of dogs followed over a longer period before any firm conclusions can be made.

## Conclusion

In conclusion, significant decreases in UP/C value occurred after one cycle of treatment with MLF and allopurinol in dogs affected with CanL. This suggests that MLF does not increase proteinuria and treatment with MLF could be considered in the management of dogs with leishmaniasis with impaired renal function at the time of diagnosis.

## Authors’ Contributions

DP designed the study. The experiment was done by DP, ES, and RP. All the authors participated in data analysis, draft, and revision of the manuscript. All authors read and approved the final manuscript.
